# Revealing key lncRNAs in cytogenetically normal acute myeloid leukemia by reconstruction of the lncRNA–miRNA–mRNA network

**DOI:** 10.1038/s41598-022-08930-6

**Published:** 2022-03-23

**Authors:** Tao Sun, Lin Dong, Yan Guo, Hai Zhao, Manzhi Wang

**Affiliations:** 1 Department of Orthopedic Surgery, The First Affiliated Hospital of Shandong First Medical University & Shandong Provincial Qianfoshan Hospital, Jinan, Shandong China; 2 Department of Hematology, The First Affiliated Hospital of Shandong First Medical University & Shandong Provincial Qianfoshan Hospital, No. 16766, Jingshi Road, Lixia District, Jinan, Shandong China; 3 Department of Pathology, The First Affiliated Hospital of Shandong First Medical University & Shandong Provincial Qianfoshan Hospital, Jinan, Shandong China

**Keywords:** Cancer, Cancer, Genetics, Biomarkers, Oncology

## Abstract

Cytogenetically normal acute myeloid leukemia (CN-AML) is a heterogeneous disease with different prognoses. Researches on prognostic biomarkers and therapy targets of CN-AML are still ongoing. Instead of protein-coding genes, more and more researches were focused on the non-coding RNAs especially long non-coding RNAs (lncRNAs) which may play an important role in the development of AML. Although a large number of lncRNAs have been found, our knowledge of their functions and pathological process is still in its infancy. The purpose of this research is to identify the key lncRNAs and explore their functions in CN-AML by reconstructing the lncRNA–miRNA–mRNA network based on the competitive endogenous RNA (ceRNA) theory. We reconstructed a global triple network based on the ceRNA theory using the data from National Center for Biotechnology Information Gene Expression Omnibus and published literature. According to the topological algorithm, we identified the key lncRNAs which had both the higher node degrees and the higher numbers of lncRNA–miRNA pairs and total pairs in the ceRNA network. Meanwhile, Gene Ontology (GO) and pathway analysis were performed using databases such as DAVID, KOBAS and Cytoscape plug-in ClueGO respectively. The lncRNA–miRNA–mRNA network was composed of 90 lncRNAs,33mRNAs,26 miRNAs and 259 edges in the lncRNA upregulated group, and 18 lncRNAs,11 mRNAs,6 miRNAs and 45 edges in the lncRNA downregulated group. The functional assay showed that 53 pathways and 108 GO terms were enriched. Three lncRNAs (XIST, TUG1, GABPB1-AS1) could possibly be selected as key lncRNAs which may play an important role in the development of CN-AML. Particularly, GABPB1-AS1 was highly expressed in CN-AML by both bioinformatic analysis and experimental verification in AML cell line (THP-1) with quantitative real‐time polymerase chain reaction. In addition, GABPB1-AS1 was also negatively correlated with overall survival of AML patients. The lncRNA–miRNA–mRNA network revealed key lncRNAs and their functions in CN-AML. Particularly, lncRNA GABPB1-AS1 was firstly proposed in AML. We believe that GABPB1-AS1 is expected to become a candidate prognostic biomarker or a potential therapeutic target.

## Introduction

Cytogenetically normal acute myeloid leukemia (CN-AML), the most common AML type, is characterized by the absence of microscopically detectable chromosome abnormalities. This is a heterogeneous disease with different prognoses. Therefore, how to classify CN-AML patients with different prognoses and give individualized treatment strategies according to the prognostic stratification will challenge our clinicians.

With the advent of high-throughput technologies, some prognostic gene expression signatures have been proposed in CN-AML. For example, patients with the FLT3 mutation always had a bad prognosis^1^, while the NPM1 or CEBPA mutations indicated a good one^2^. Prognostic genes proposed so far mainly focused on protein-coding genes. However, protein-coding genes only account for approximately 1.5% of the whole genome, which means more than 98% of the human genome does not encode protein sequences^3^. Therefore, we still need to explore the prognostic hallmarks and possible therapeutic targets in CN-AML, especially in terms of non-coding RNAs. Recently, long non-coding RNA (lncRNA) has attracted much attention since increasing evidences indicated that they played critical roles in multiple biological processes, such as cell differentiation, immune response, cell cycle control, imprinting and splicing, based on diverse underlying mechanisms^4–6^. Because of the complex and powerful functions of lncRNAs, it is not surprise that mutations and dysregulations of these lncRNAs are associated with the development and progression of various complex human diseases such as cancers, Alzheimer’s diseases, cardiovascular diseases, diabetes, and neurodegeneration diseases^7,8^. Notably, lncRNAs may also play an important role in pathogenesis of AML. For example, lncRNA NEAT1 repressed the expression of miR-23a-3p and therefore modulated cell proliferation and apoptosis in AML by regulating SMC1A^9^. Furthermore, Garzon et al. built a prognostic lncRNA score system for older patients (> 60 years) with cytogenetically normal AML^10^. Therefore, exploring the functions of CN-AML associated lncRNAs will provide potential biomarkers for AML diagnosis, treatment and prognosis.

Noteworthy, competing endogenous RNA (ceRNA) network is a promising module to facilitate lncRNAs function in complex pathologic conditions. Salmena et al.^11^ has suggested that all types of RNA transcripts (mRNA, pseudogenes, lncRNA, etc.) can crosstalk with each other by competing for miRNAs through shared miRNA-binding sites [‘miRNA response elements’ (MREs)], and outlined this ceRNA hypothesis which has been confirmed by many experimental evidences^9,12,13^. So lncRNAs can regulate the expression of target genes by binding and sequestering gene associated miRNAs. CeRNAs can be found in all organisms that use miRNAs to regulate gene expression. Given the prominent functions of ceRNAs in physiology, unbalanced ceRNAs can also promote tumorigenesis and progression, and greatly contribute to tumor risk classification and prognosis. Until now, there are many bioinformatic analyses of the ceRNA network in AML. For example, Yaqi Cheng et al. established an AML prognostic circRNA-lncRNA-miRNA-mRNA ceRNA regulatory network based on 6 prognostic hub mRNAs^14^. Nan Zhang et al. constructed a lncRNA-mRNA-miRNA ceRNA network in childhood AML by comparing gene expression differences between high-risk and low-risk patients^15^. Xuejiao Yin et al. constructed a survival specific ceRNA network in pediatric and adolescent CN-AML^16^. But no research has directly compared the differential expression of RNAs and established the ceRNA networks between CN-AML patients and normal individuals.

**Figure 1:**
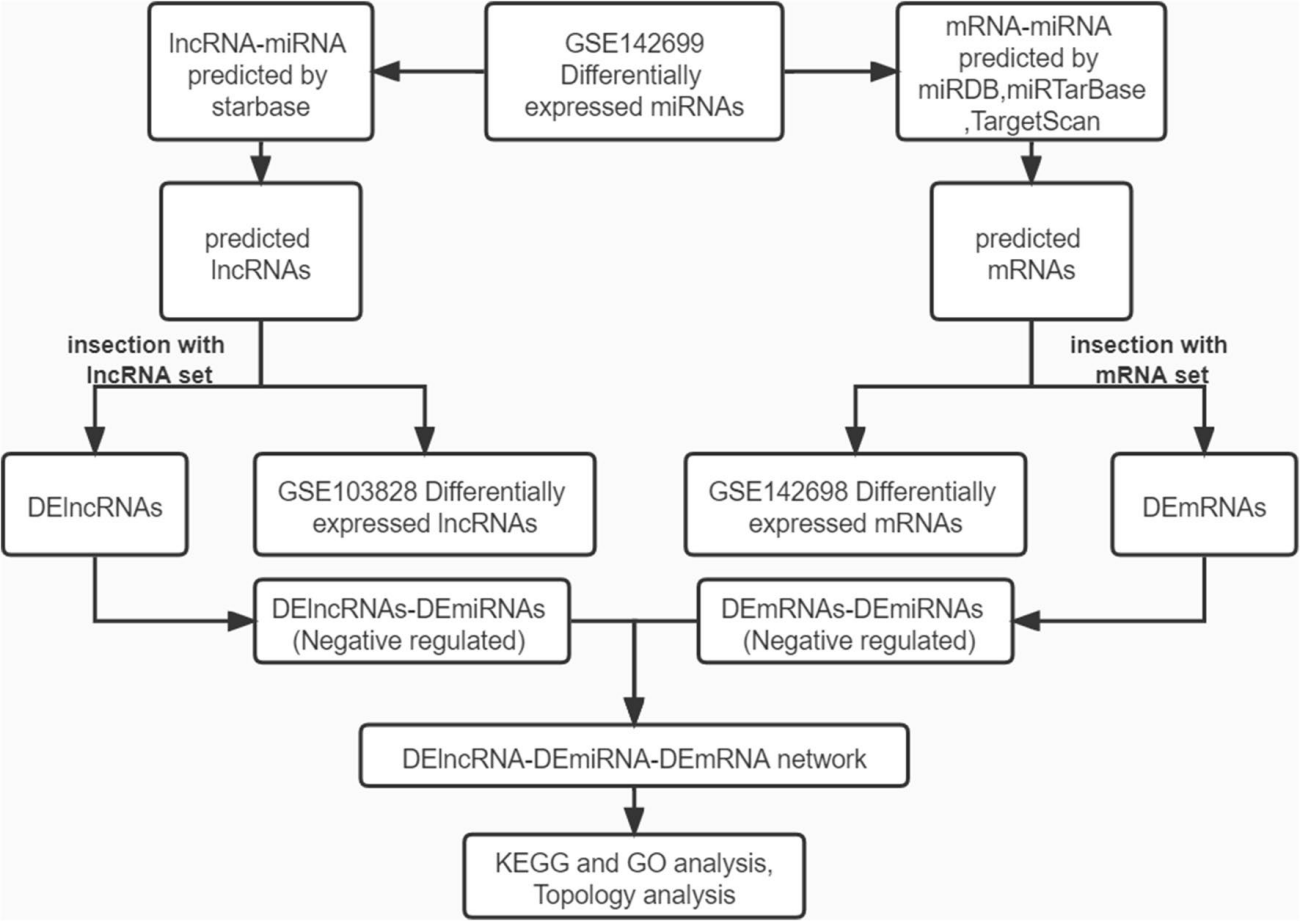
The flowchart for reconstruction of the lncRNA–miRNA–mRNA network. First, miRNA/lncRNA/mRNA expression data were downloaded from NCBI GEO. Second, differentially expressed miRNAs (DEmiRNAs), lncRNAs (DElncRNAs) and mRNAs (DEmRNAs) were screened. Third, target mRNAs of DEmiRNAs were predicted using miRDB, miRTarBase and TargetScan databases. And target lncRNAs of DEmiRNAs were predicted using starbase database. Then, DElncRNAs and DEmRNAs were merged with the target lncRNAs and mRNAs of DEmiRNAs, respectively. The co-expression lncRNAs and mRNAs were selected. Finally, the DEmiRNAs, co-expression lncRNAs and mRNAs were mapped into the interactions.

So, in order to further explore the functional lncRNAs in CN-AML, we intended to compare CN-AML patients with normal controls directly, to find out the differentially expressed lncRNAs, miRNAs, and mRNAs, and built a global triple network based on the ceRNA theory (Fig. 1).

## Results

### Differentially expressed lncRNA, miRNA, and mRNA in CN-AML

**Figure 2:**
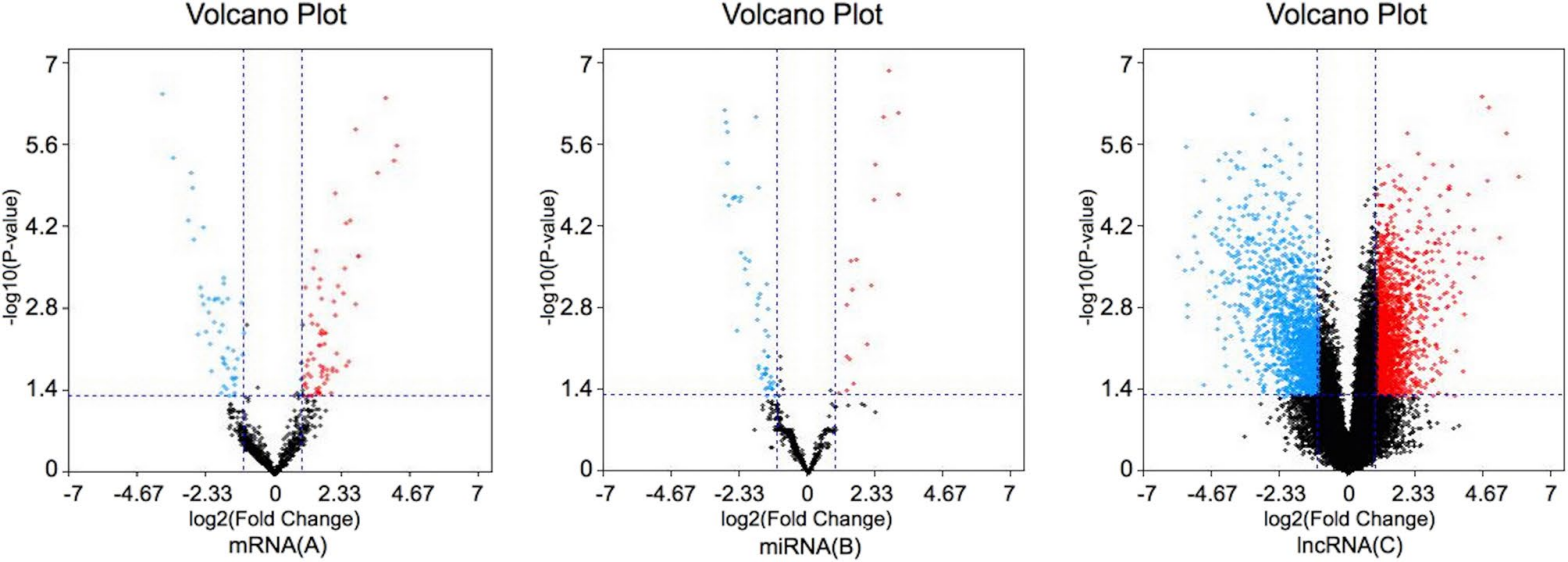
Volcano plots reflecting number, significance and reliability of differentially expressed RNAs in CN-AML compared with normal controls. The red dots indicate upregulation and blue dots indicate downregulation of mRNAs (**A**), miRNAs (**B**) and LncRNAs (**C**). The x-axis represents the value of log2 (Fold Change) and the y-axis represents the value of -log10 (*p* value).

A total of 127 mRNAs, 3380 lncRNAs, and 82 miRNAs were found to be differentially expressed in CN-AML group compared with normal controls (|log2fold change|> 1 and adj. *P* value < 0.05 as the standards), of which 76 mRNAs (59.84%), 1751 lncRNAs (51.80%) and 19 miRNAs (23.17%) were upregulated while others were downregulated. Volcano plots, visually demonstrating the distribution of RNAs, were shown in Fig. 2.

### Reconstruction of the lncRNA–miRNA–mRNA network

**Figure 3:**
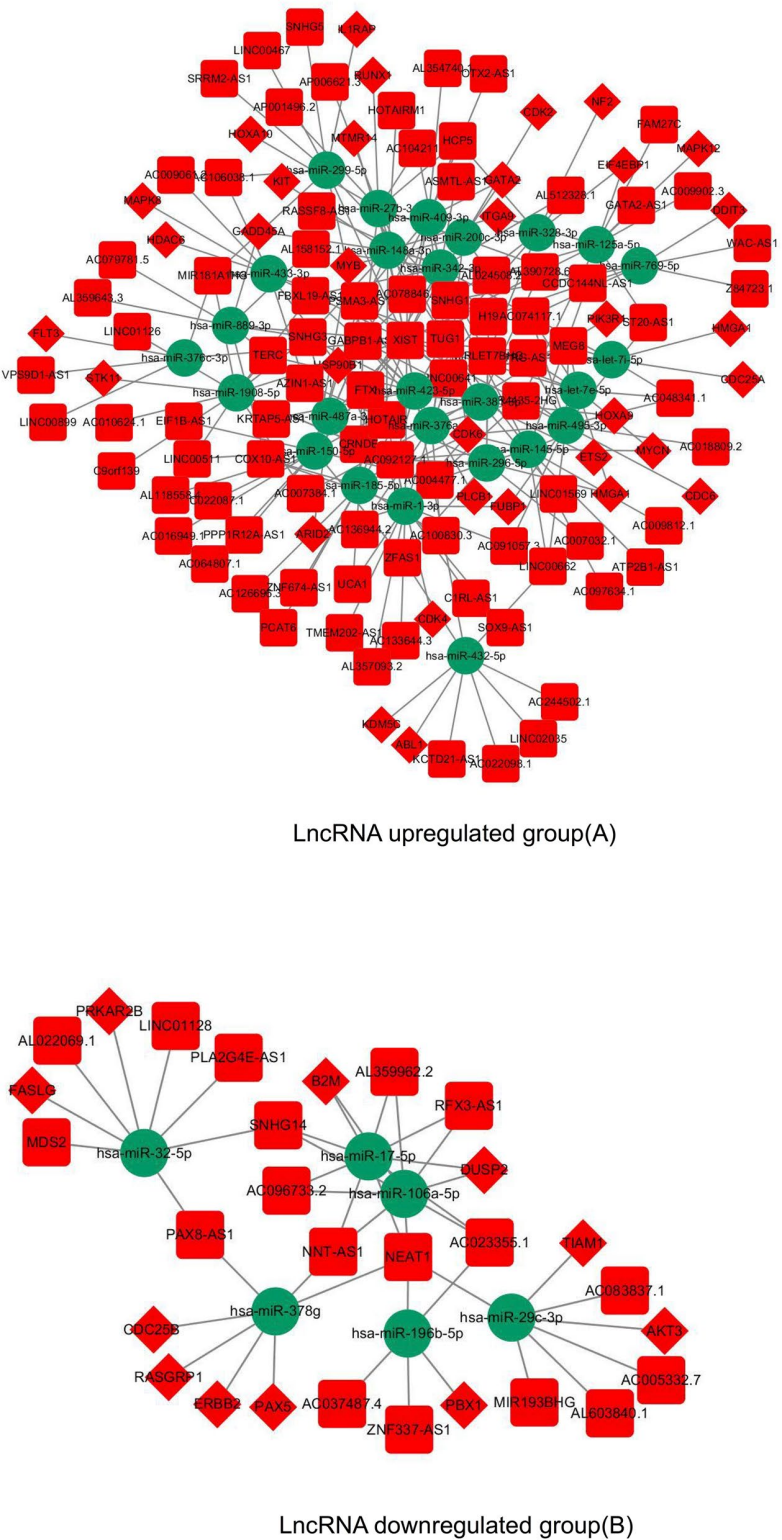
The view of the lncRNA–miRNA–mRNA network in CN-AML. The square represents lncRNAs, the rhombus represents mRNAs, and the circle represents miRNAs. There were 90 lncRNAs, 33mRNAs, 26 miRNAs and 259 edges in the lncRNA upregulated group (**A**), and 18 lncRNAs, 11 mRNAs, 6 miRNAs and 45 edges in the lncRNA downregulated group (**B**).

To speculate on the functions of lncRNAs acting as miRNA targets, a network among lncRNAs, miRNAs, and mRNAs was reconstructed and then visualized. As shown in Fig. 3, there were 90 lncRNAs, 33mRNAs, 26 miRNAs and 259 edges in the lncRNA upregulated group, and 18 lncRNAs, 11 mRNAs, 6 miRNAs and 45 edges in the lncRNA downregulated group.

### Topological analysis of the CN-AML related lncRNA–miRNA–mRNA network

Han et al.^17^ have defined the hub nodes which play critical roles in biological networks as node degrees exceeding 5. So by calculating all node degrees of the lncRNA–miRNA–mRNA network, we found that 42 nodes could be chosen as hub nodes, including 10 lncRNAs, 31 miRNAs, and 1 mRNA (CDK6). Moreover, the number of the first relationship pairs of lncRNA–miRNA and the secondary relationship pairs of miRNA–mRNA were calculated. The results were shown in Table 1. Interestingly, we found that three lncRNAs (XIST, TUG1, GABPB1-AS1) not only had higher node degrees, but also had higher numbers of lncRNA–miRNA pairs and total pairs. It suggested that this three lncRNAs may play crucial roles in the development of CN-AML, which could be selected as key lncRNAs.

**Table 1:** The topological algorithm of differentially expressed lncRNAs (Top10).

Number	Gene name	Node degree	lnc-miRNA pairs	mi-mRNA pairs	Total
1	XIST	20	20	38	58
2	TUG1	10	10	23	33
3	GABPB1-AS1	10	10	13	23
4	MIRLET7BHG	7	7	11	18
5	AC092127.1	6	6	12	18
6	SNHG1	6	6	9	15
7	NEAT1	5	5	11	16
8	SNHG3	5	5	9	14
9	H19	5	5	9	14
10	AC074117.1	5	5	9	14

### Functional annotation of the lncRNA–miRNA–mRNA network

To explore the biological processes and pathways of differentially expressed mRNAs (DEmRNAs) in the lncRNA–miRNA–mRNA network, we conducted Gene Ontology (GO) term and Kyoto Encyclopedia of Genes and Genomes (KEGG) pathway analysis.

**Figure 4:**
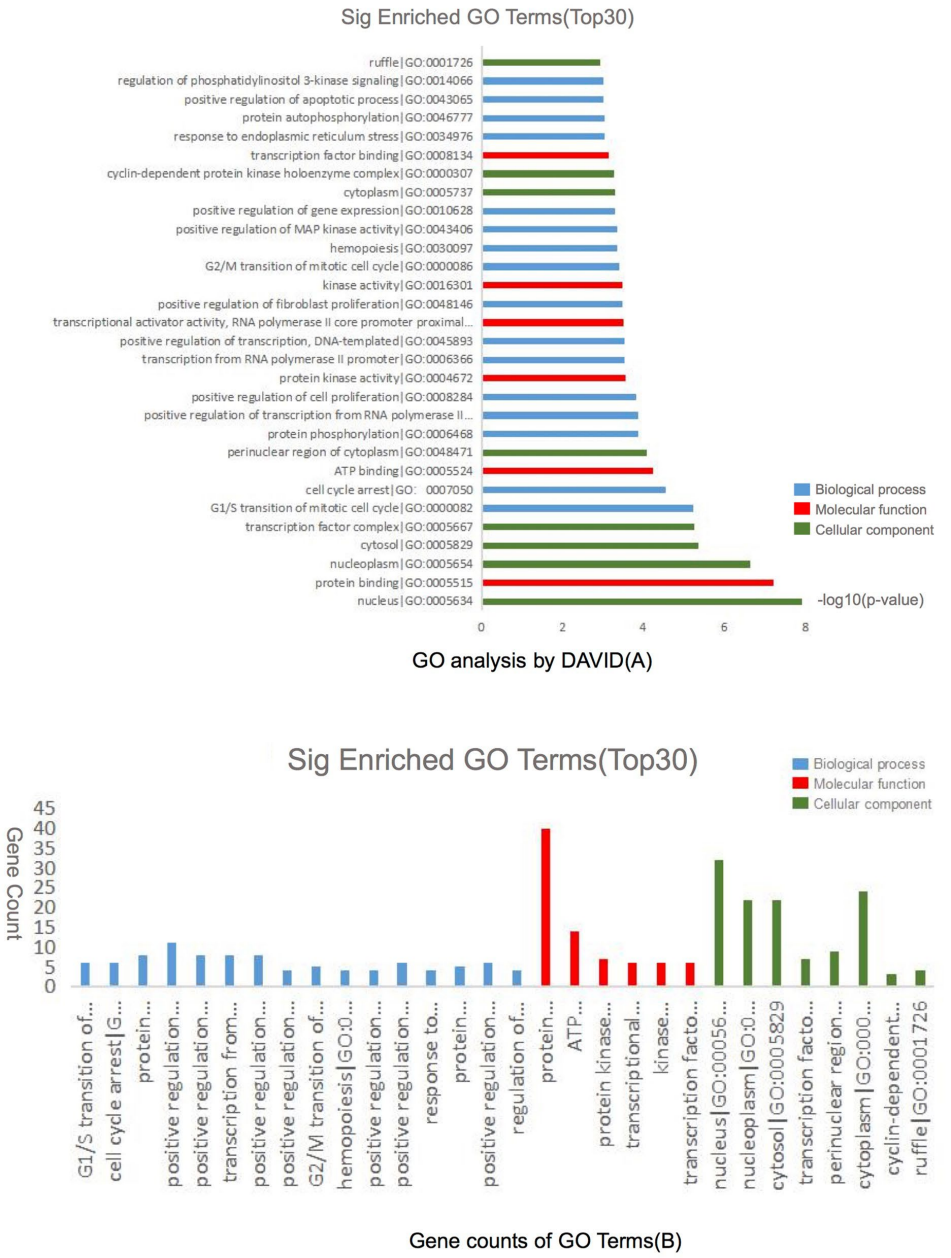
KEGG pathway analysis of DEmRNAs in the lncRNA–miRNA–mRNA network. (**A**) The top20 significantly enriched pathway terms by KOBAS. (**B**) The KEGG pathway interactions by Cytoscape plug-in ClueGO. Genes RUNX1, FLT3, KIT, FASLG, AKT3, MAPK8, GADD45A and PIK3R1 were enriched in pathways greater than two terms by Cytoscape plug-in ClueGO.

The DEmRNAs were classified into three functional groups: biological process group, molecular function group, and cellular component group. As shown in Fig. 4, in the biological process group, DEmRNAs mainly enriched in G1/S transition of mitotic cell cycle, cell cycle arrest and protein phosphorylation. In the molecular function group, DEmRNAs mainly enriched in protein binding, ATP binding and protein kinase activity. In the cellular component group, DEmRNAs mainly enriched in nucleus, nucleoplasm and cytosol.

**Figure 5:**
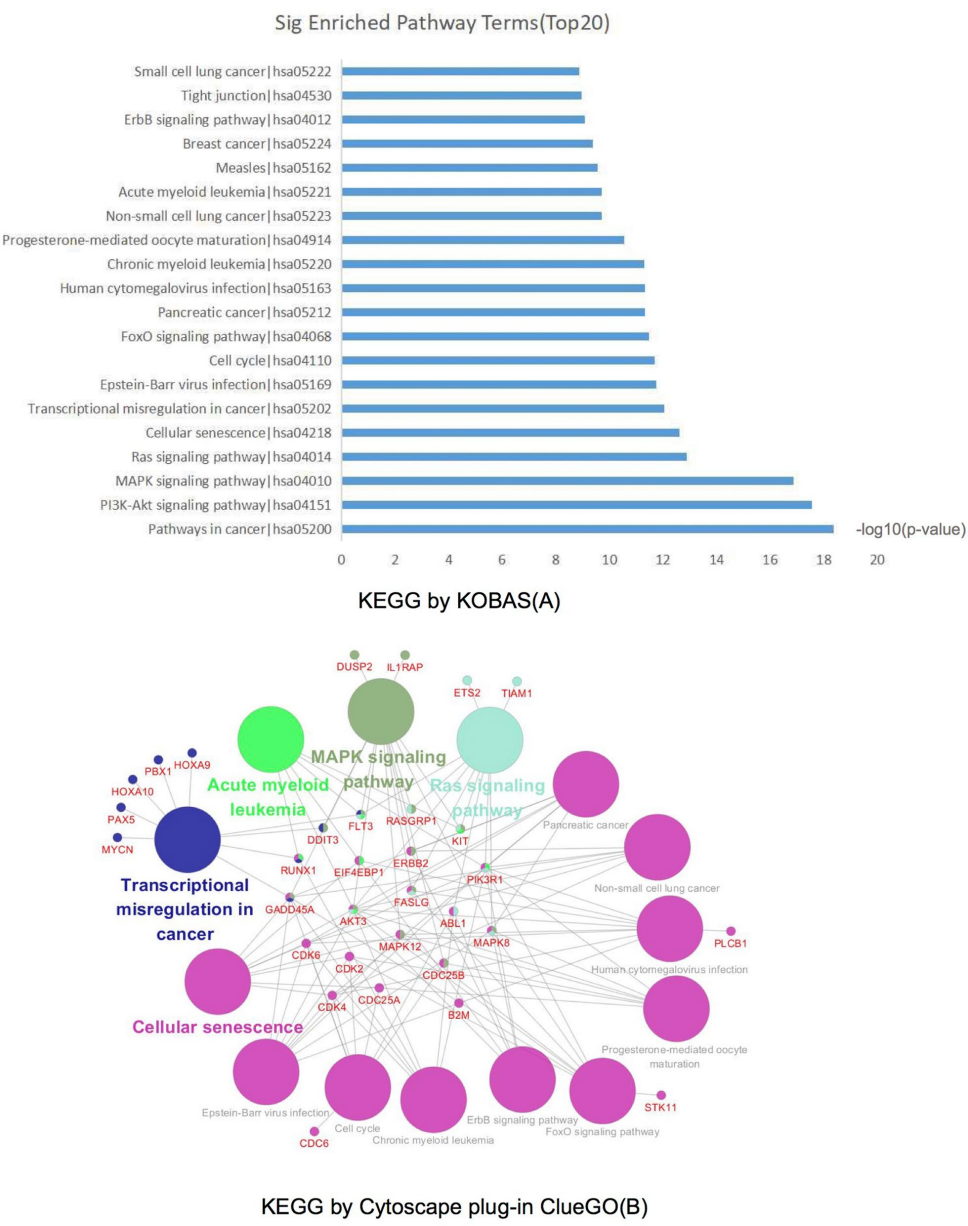
Gene Ontology analysis and significantly enriched GO terms of DEmRNAs in the lncRNA–miRNA–mRNA network. (**A**) Significantly enriched GO terms of DEmRNAs in the lncRNA–miRNA–mRNA network based on their functions. (**B**) GO analysis classified DEmRNAs in the lncRNA–miRNA–mRNA network into 3 groups (i.e., molecular function, biological process and cellular component).

The KEGG analysis revealed the potential biological functions (*p* value < 0.05). A total of 53 significantly enriched pathways were obtained. Among these pathways, ‘PI3K-Akt signaling pathway’^18^, ‘Ras signaling pathway’^19^, ‘MAPK signaling pathway’^20^, ‘FoxO signaling pathway’^21^, were related with the development of AML. Additionally, some other pathways such as ‘Pathways in cancer’^22^, ‘ErbB signaling pathway’^23^ were also tumor related pathways. Genes RUNX1, FLT3, KIT, FASLG, AKT3, MAPK8, GADD45A and PIK3R1 were enriched in greater than or equal to three pathway terms. The mentioned pathways were showed in Fig. 5.

### Reconstruction of the key lncRNA–miRNA–mRNA sub-networks

**Figure 6:**
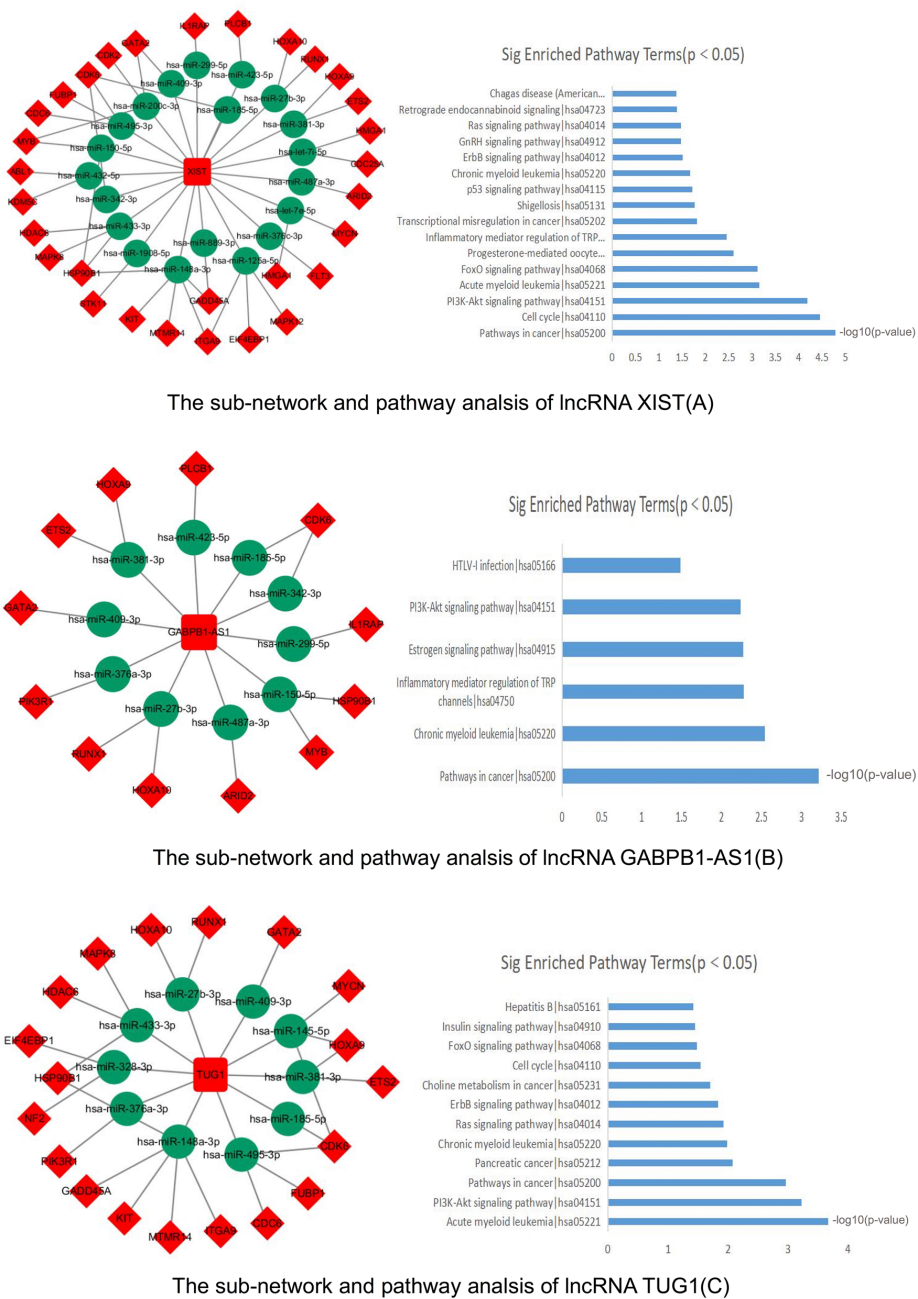
The sub-networks of three key lncRNAs and their KEGG pathway analysis by KOBAS. (**A**) The sub-network of lncRNA XIST and the significantly enriched pathway terms of its related mRNAs. (**B**) The sub-network of lncRNA GABPB1-AS1 and the significantly enriched pathway terms of its related mRNAs. (**C**) The sub-network of lncRNA TUG1 and the significantly enriched pathway terms of its related mRNAs.

The key three lncRNAs (XIST, TUG1, GABPB1-AS1) and their linked miRNAs/mRNAs were extracted and used to reconstruct the new subnetworks as follows (Fig. 6). Pathway analysis showed that lncRNA XIST related mRNAs were significantly enriched in 16 pathway terms including ‘PI3K-Akt signaling pathway’, ‘FoxO signaling pathway’, ‘p53 signaling pathway’^24^ and ‘Ras signaling pathway’, all of which have been shown to play important roles in AML. LncRNA TUG1 related mRNAs were significantly enriched in 12 pathway terms including ‘PI3K-Akt signaling pathway’, ‘FoxO signaling pathway’, and ‘Ras signaling pathway’, all of which have also been shown to play important roles in AML. LncRNA GABPB1-AS1 related mRNAs were significantly enriched in 6 pathway terms including ‘PI3K-Akt signaling pathway’ and ‘Pathways in cancer’.

### Survival analysis of the key RNAs

**Figure 7:**
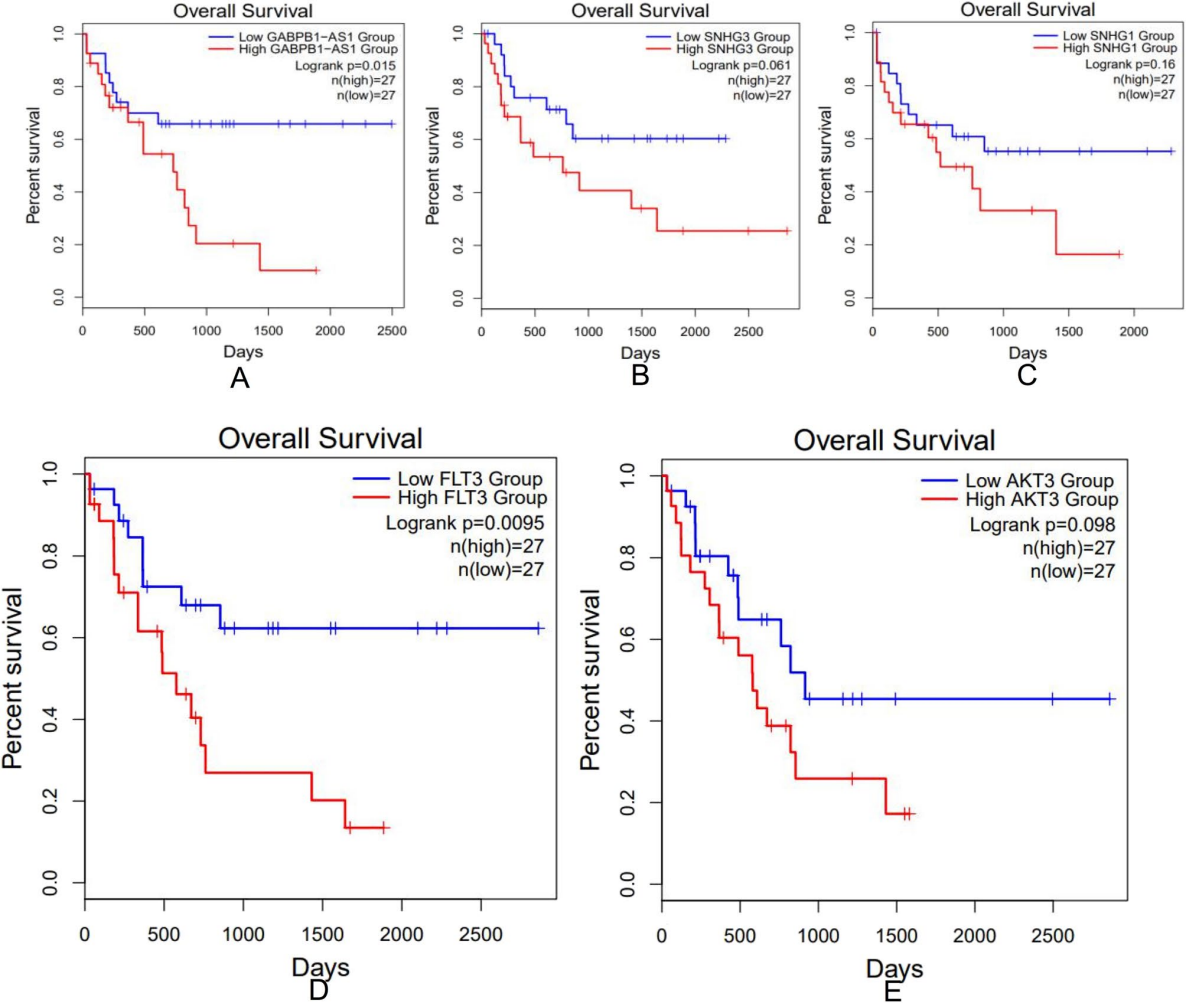
Kaplan–Meier curves of three lncRNAs (GABPB1-AS1 (**A**), SNHG3 (**B**), SNHG1 (**C**)) and two mRNA (FLT3 (**D**), AKT3 (**E**)) in AML with logrank *p* < 0.05.

We conducted the survival analysis of the key lncRNAs (showing in Table 1) and key mRNAs (showing in Fig. 5B) involved in the lncRNA–miRNA–mRNA network by Kaplan–Meier curve with *P* value < 0.05. Only three lncRNAs (GABPB1-AS1, SNHG3, SNHG1) and two mRNA (FLT3, AKT3) were significantly related to overall survival (OS) of AML patients (Fig. 7).

### The expression of GABPB1-AS1 in AML by quantitative real‐time polymerase chain reaction (qRT-PCR)

**Figure 8:**
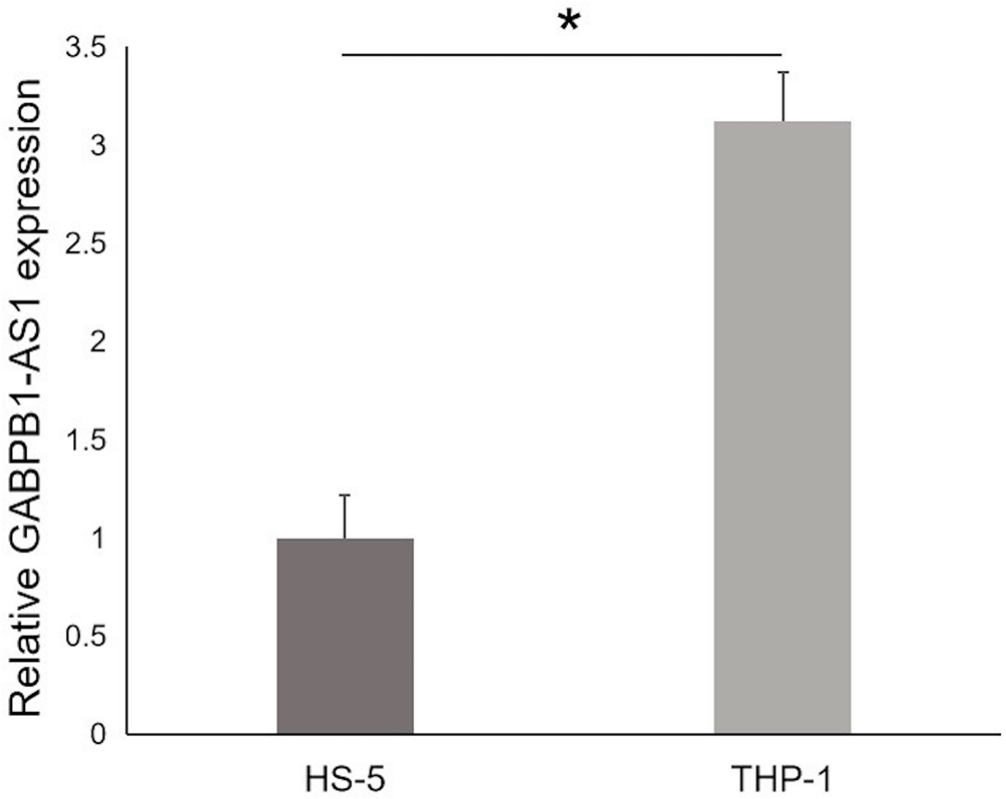
Comparing differences in the expression levels of GABPB1-AS1 between THP-1 cells and normal HS-5 cells. *p < 0.01.

To validate the above results of bioinformatic analysis, we conducted qRT-PCR to detect the expression of lncRNA GABPB1-AS1 in AML cell line (THP-1). GABPB1-AS1 which took the central place in the lncRNA–miRNA–mRNA network was significantly high expressed in THP-1 cells compared with HS-5 cells (control cell line) (*p* < 0.01) (Fig. 8).

## Discussion

Acute myeloid leukemia (AML) is the most common type of acute leukemia in adults. According to the WHO classification, patients with recurring cytogenetic abnormalities, such as t (8,21), inv (16) (p13q22) or t (15,17) often had a better prognosis^25^. But for those with normal karyotype, there is an another story. Actually, CN-AML is a heterogeneous disease. Although with the advent of high-throughput sequencing and other methods, many genetic changes (such as mutations of gene FLT3, NPM1) which were closely related to the prognosis of CN-AML have been discovered, but the pathogenesis and prognostic markers of CN-AML were not yet fully understood. In recent years, more and more studies have focused on the epigenetic regulation of AML. Non-coding RNA (such as lncRNA or miRNA) is an important part of the epigenetic regulation. Recent studies have shown that lncRNAs are closely related to tumor cell proliferation, invasion, metastasis, apoptosis and tumor angiogenesis^26^. However, the specific functions of lncRNAs in CN-AML are still unclear.

The ceRNA network is a complex post-transcriptional regulatory network using MREs to compete for the binding of miRNAs thereby implementing mutual control between mRNAs, lncRNAs and miRNA^27^. Thus, it has been shown that an efficient way to infer the potential functions of lncRNAs is by studying their relationship with miRNAs and/or mRNAs, whose functions have been annotated. Therefore, we can use the ceRNA network to explore the specific functional roles and prognostic values of lncRNAs in CN-AML.

In this study, we conducted the differential expression analysis between CN-AML patients and normal controls to find differentially expressed RNAs by Gene Expression Omnibus (GEO) data sets (GSE142699, GSE142698, GSE103828) and literature review. Then the lncRNAs/mRNAs targeted by miRNAs were searched through the online databases. Afterwards, we took the intersection between the targeted RNAs with the above-mentioned differentially expressed RNAs to obtain DElncRNA–DEmiRNA and DEmiRNA–DEmRNA pairs, in which the expression of DEmiRNAs were negatively correlated with both DElncRNAs and DEmRNAs. Finally, a global triple network of CN-AML was constructed.

It’s worth noting that these differentially expressed genes were not unique to CN-AML. For example, abnormalities in the gene FLT3 are also found in some APL patients with t(15,17) ^28^, and may be associated with poor prognosis. Abnormalities in gene c-kit^29^ and gene RUNX1^30^ can also be seen in some AML patients with t(8,21) or inv(6). The occurrence of AML is usually the result of multiple hits, and the phenomenon of cytogenetic abnormalities combined with molecular abnormalities is very common. In addition, there may also be multiple genetic abnormalities in one patient. Under different cytogenetic backgrounds, the types and frequencies of abnormal genes are not the same, so we need to further study the molecular changes in different types of AML and explore their pathogenic values.

We conducted KEGG and GO analyses of the DEmRNAs in the lncRNA–miRNA–mRNA network. The KEGG analysis showed that 29 pathways were significantly enriched (*P* value < 0.05). ‘PI3K-Akt signaling pathway’, ‘MAPK signaling pathway’ and ‘Ras signaling pathway’, which have been shown to play important roles in AML, were involved. Furthermore, 108 GO terms were significantly enriched with *P* value < 0.05. These significant GO terms involved G1/S transition of mitotic cell cycle, cell cycle arrest and protein phosphorylation for the biological process group; protein binding, ATP binding and protein kinase activity for the molecular function group; and nucleus, nucleoplasm and cytosol for the cellular component group.

In order to find the key lncRNAs, which can be used as potential biomarkers for clinical diagnosis and treatment targets of CN-AML, the hub nodes and the number of relationship pairs were counted. In this study, three lncRNAs (XIST, TUG1, GABPB1-AS1) were found to be topological hub nodes whose node degrees and the numbers of lncRNA–miRNA pairs and total pairs were significantly higher compared to other lncRNAs. This indicated that these lncRNAs had profound implications for CN-AML, and can be considered as key lncRNAs.

### LncRNA XIST

The XIST locus produces a 17–20 kb RNA that coats the X chromosome in cis, and plays an essential role in X chromosome inactivation^31^. Recently, XIST has been reported to function as an oncogene or a tumor suppressor in different human malignancies^32^. For example, XIST could act as an oncogene in breast cancer and was closely associated with a poor prognosis^33^. Down-regulation of XIST has been reported to reduce chemo-resistance in non-small cell lung cancer cells by inhibiting autophagy^34^. In AML, XIST was highly expressed in patients’ bone marrow cells. In addition, silencing XIST could repress AML bone marrow cell proliferation while enhance apoptosis and adriamycin sensitivity of AML cells^35^.

In this study, lncRNA XIST was highly expressed in CN-AML and had the highest node degrees and the highest number of lncRNA–miRNA pairs and total pairs in the ceRNA network among all the lncRNAs. This means XIST may play an important role in CN-AML which was consistent with previous findings mentioned above. The pathway analysis of key lncRNA XIST–miRNA–mRNA sub-network showed that 16 pathways were significantly enriched. ‘PI3K-Akt signaling pathway’, ‘FoxO signaling pathway’, ‘p53 signaling pathway’ and ‘Ras signaling pathway’, which have been shown to play important roles in AML, were involved. In addition, ‘PI3K-Akt signaling pathway’^36^, ‘cell cycle’^37^, and ‘acute myeloid leukemia’^35^ pathways have been experimentally confirmed to be related with XIST.

### LncRNA TUG1

LncRNA Taurine-Upregulated Gene1 (TUG1), located on chromosome 22q12, a critical oncogenic lncRNA of human, has been proved to take part in hematological cancers. Interestingly, Some scholars demonstrated that TUG1 was highly expressed in tissues and cell lines of AML patients, and the high expression of TUG1 was also closely related to poor prognosis of AML^38^. TUG1 induced cell proliferation, and restrained cell apoptosis in AML by targeting aurora kinase A^39^. TUG1 facilitated the cell viability and metastasis by targeting miR-370-3p/MAPK1/ERK in AML^40^. In addition, TUG1 silencing decreased the IC50 of adriamycin, and promoted adriamycin-induced apoptosis in AML cells by miR-34a/EZH2 axis^41^, providing a potential therapeutic target for AML.

In our study, lncRNA TUG1 was highly expressed in CN-AML and had the higher topological parameters in the ceRNA network. This means TUG1 may play an important role in CN-AML which was consistent with previous findings mentioned above. The pathway analysis of key lncRNA TUG1–miRNA–mRNA sub-network showed that 12 pathways were enriched. ‘PI3K-Akt signaling pathway’, ‘FoxO signaling pathway’, and ‘Ras signaling pathway’, which have been shown to play important roles in AML, were involved. In addition, ‘PI3K-Akt signaling pathway’^42^, ‘cell cycle’^43^, ‘acute myeloid leukemia’^44^ and ‘Insulin signaling pathway’^45^ pathways have been experimentally confirmed to be related with TUG1.

### LncRNA GABPB1-AS1

LncRNA GAbinding protein transcription factor subunit beta-1 antisense RNA 1 (GABPB1-AS1) is the antisense RNA of GABPB1 mRNA, which is located in the cytoplasm and has a total length of 4139nt^46^. GABPB1-AS1 was identified for the first time in human-induced pluripotent stem cells (hiPSCs). The expression level of GABPB1-AS1 is increased in hiPSCs under the chemical stresses (cadmium, hydrogen peroxide, and cycloheximide)^47^. LncRNA GABPB1-AS1 also played a role in several cancers. But the results were contradictory. As a tumor suppressor gene, Qi et al. found that GAPBPB1-AS1 inhibited the antioxidant ability of hepatocellular carcinoma cancer cells and cell proliferation by inhibiting the expression of GABPB1 and peroxiredoxin 5^48^. In addition, GABPB1-AS1 inhibited clear cell renal cell carcinoma growth and played a tumor suppressor role through an miR-1246/PCK1 axis^49^. As a tumor activator gene, GABPB1-AS1 can bind to miR-519e-5p and destroy its tumor suppressive function in pathogenesis of cervical cancer^50^. Furthermore, findings suggested that the decrease in GABPB1-AS1 expression associated with decreased breast cancer risk^51^. Alkhateeb et al. also revealed that the aberrant expression of GABPB1-AS1 can be used as a potential biomarker of prostate cancer^52^. However, the role of GABPB1-AS1 in AML is still unclear.

In this study, for the first time, we came to a conclusion that lncRNA GABPB1-AS1 was highly expressed in CN-AML by both bioinformatic analysis and qRT-PCR verification in AML cell line (THP-1). The pathway analysis of key lncRNA GABPB1-AS1–miRNA–mRNA sub-network showed that 6 pathways were significantly enriched and primarily involved ‘PI3K-Akt signaling pathway’ and ‘Pathways in cancer’ pathway terms. In addition, the survival analysis told us that patients with lower expression of GABPB1-AS1 had better prognosis. In conclusion, GABPB1-AS1 would like to be a potential prognostic marker and a therapeutic target of CN-AML.

Until now, the researches of lncRNAs’ functions in diseases are still in its infancy. Facing lack of standardization and information redundancy among different databases, a more comprehensive method for extraction and integration of data is urgently needed^8^. And the identification of lncRNA and miRNA associations in large volumes of data remains difficult as well^53^. In recent years, different computational models have been proposed to handle these problems with the development of gene sequencing technology and the acquisition of plenty of gene expression data^54^. For example, based on the assumption that similar diseases tend to be associated with functionally similar lncRNAs, Chen et al. has developed a computational model of Laplacian Regularized Least Squares for LncRNA–Disease Association (LRLSLDA) in the semisupervised learning framework to identify potential disease–lncRNA associations by integrating known associations and lncRNA expression profiles^55^. Subsequently, the biological network-based model of KATZ measure for LncRNA-Disease Association prediction (KATZLDA) was proposed and achieved better prediction performance^56^. In 2021, Li Zhang et al. designed a more reliable model based on the interactome network and graphlet interaction (LMI-INGI), which could effectively predict potential lncRNA–miRNA interactions^57^. Subsequently, based on distance analysis of an integrated network of the sequence similarity networks of lncRNAs and miRNAs and the Gaussian interaction profile (GIP) kernel similarity network, a new NDALMA model was built, showing efficient and feasible outcomes^53^. In this study, we combined the ceRNA theory with bioinformatic analysis, trying to establish the lncRNA-miRNA-mRNA network in CN-AML and identify the key RNAs through the topological algorithm of the triple network. The models mentioned above should be effective ways to enrich the ceRNA network in the future study. The functions of lncRNAs are very complex, and the triple network established based on the ceRNA theory only revealed a small part of the regulating functions of lncRNAs. We believe that with the further development of computer technology, more computer models such as the ones mentioned above will be applied to explore the functions of lncRNAs, and provide more possibilities for further experimental researches and clinical translation.

In summary, our study constructed a lncRNA–miRNA–mRNA network associated with CN-AML, and explored novel lncRNAs (especially GABPB1-AS1) as potential diagnostic and prognostic biomarkers. The specific functions of these key lncRNAs in CN-AML need further experimental verification.

## Conclusion

Based on the ceRNA theory, we reconstructed a lncRNA– miRNA–mRNA network of CN-AML by comparing with normal controls for the first time. According to the topological algorithm, our study further found that three lncRNAs (XIST, TUG1, GABPB1-AS1) could possibly be selected as key lncRNAs which may play an important role in the development of CN-AML. Particularly, lncRNA GABPB1-AS1 was firstly proposed in AML. GABPB1-AS1 was highly expressed in CN-AML by both bioinformatic analysis and qRT-PCR verification in AML cell line (THP-1). In addition, it was also negatively correlated with OS of AML patients. So GABPB1-AS1 is expected to become a candidate diagnostic biomarker or a potential therapeutic target. In addition, we conducted the functional analyses of the ceRNA network to improve our understanding of the pathogenesis of CN-AML from the perspective of lncRNAs. Further studies are needed to verify the biological functions and molecular mechanisms of these specific lncRNAs in CN-AML.

## Methods

### Raw data

GEO is a public functional genomics data repository supporting MIAME-compliant data submissions. Array- and sequence-based data were accepted.

In order to find the differentially expressed miRNAs, mRNAs and lncRNAs in CN-AML compared with normal controls, databases (GSE142699, GSE142698 and GSE103828) were downloaded respectively from NCBI GEO.

In addition, we also searched in pubmed by keywords ‘acute myeloid leukemia and lncRNA’ to find lncRNAs which have been reported to have significantly differential expressions in CN-AML.

The RNA-seq data and clinical data are publicly available on open-access. Therefore, no further approval was required from the local ethics committee.

### Screening differentially expressed lncRNAs, miRNAs and mRNAs

The ‘edgeR’ package^58^ which is a Bioconductor software package for examining differential expression of replicated count data was utilized to identify the differentially expressed RNAs in CN-AML compared with normal controls. In this methodology, an overdispersed Poisson model is used to account for both biological and technical variability. Empirical Bayes methods are used to moderate the degree of overdispersion across transcripts, improving the reliability of inference. The ‘edgeR’ package can be used even with the most minimal levels of replication. In this study, the downloaded data were calibrated, standardized and analyzed for differences to obtain differentially expressed lncRNA, miRNA, and mRNA molecules between the CN-AML group and normal control. The screening criteria of the three kinds of dysregulated RNAs were as follows: adj. *p* value < 0.05 and |log2fold change|> 1.

For lncRNAs, differentially expressed lncRNAs also includes the ones obtained from the published literature.

### Prediction of target lncRNAs and mRNAs of differentially expressed miRNAs

In this study, starbase website^59^ was used to predict lncRNA-miRNA interactions. In addition, the online websites miRDB^60^, miRTarBase^61^, TargetScan^62^ were used to predict target genes. In TargetScan, predicted targets are ranked according to the predicted efficacy of targeting as calculated using cumulative weighted context++scores of the sites, and scores < − 0.1 were selected. Genes that appeared in at least two databases or more were regarded as target genes.

### Reconstruction of the lncRNA–miRNA–mRNA network

To further improve the reliability of bioinformatic analysis, we obtained the portion of the target mRNAs or lncRNAs that overlapped with the differentially expressed mRNAs or lncRNAs in CN-AML, and overlapping RNAs were further analyzed as differentially expressed mRNAs (DEmRNAs) or differentially expressed lncRNAs (DElncRNAs). Finally, we established matched DElncRNA–DEmiRNA pairs and DEmiRNA–DEmRNA pairs.

The lncRNA–miRNA–mRNA network was reconstructed based on ceRNA theory as follows^63^: For a given co-expressed lncRNA–mRNA pair, both lncRNA and mRNA in this pair were targeted and co-expressed negatively with a certain common miRNA, and this lncRNA–miRNA–mRNA was identified as the co-expression competing triplet. The lncRNA–miRNA–mRNA network was reconstructed by assembling all co-expression competing triplets, which were identified above, and was visualized using Cytoscape software. Cytoscape software is an open source software project for integrating biomolecular interaction networks with high-throughput expression data and other molecular states into a unified conceptual framework. It also provides basic functionality to layout and query the network; to visually integrate the network with expression profiles, phenotypes, and other molecular states; and to link the network to databases of functional annotations^64^. Simultaneously, all node degrees of the lncRNA–miRNA–mRNA network were calculated.

### Functional enrichment analysis

To assess functional enrichment, GO term and KEGG pathway analyses of mRNAs in the lncRNA–miRNA– mRNA network were performed using multiple online databases, including DAVID (Database for Annotation, Visualization, and Integration Discovery)^65^ and KOBAS^66^ with *p* < 0.05 as the cut-off criterion. Then the KEGG pathway interaction network was reconstructed using Cytoscape plug-in ClueGO.

### Reconstruction of the key lncRNA–miRNA–mRNA sub-networks

Every lncRNA, its linked miRNAs and mRNAs in the global triple network were extracted and used to reconstruct the new sub-network using Cytoscape software. Meanwhile, the numbers of the first relationship pairs of lncRNA–miRNA and the secondary relationship pairs of miRNA–mRNA were calculated. Using the node degrees of lncRNAs, the numbers of the first relationship pairs of lncRNA–miRNA plus the secondary relationship pairs of miRNA–mRNA (total pairs), the key lncRNAs were verified. For further analysis, we performed pathway analyses of the individual key lncRNAs by using their first mRNA neighbors in the key lncRNA–miRNA–mRNA sub-networks^63^.

### Survival analysis

To explore the relationship between prognosis and the key lncRNAs, miRNAs and mRNAs involved in the ceRNA network, Kaplan–Meier curve were carried out at a *P* value < 0.05 using the online websites GEPIA2 (Gene Expression Profiling Interactive Analysis), which is an updated and enhanced version for gene expression analysis based on tumor and normal samples from the TCGA and the GTEx databases. Featuring 198,619 isoforms and 84 cancer sub-types, GEPIA2 has extended gene expression quantification from the gene level to the transcript level, and supports analysis of a specific cancer sub-type, and comparison between sub-types^67^.

### RNA extraction, reverse transcription (RT), and quantitative real‐time polymerase chain reaction (qRT-PCR)

The total RNAs were extracted using TRIzol kits (Pufei, Shanghai, China) for qRT-PCR analyses. Reverse transcription was then conducted by applying the Promega RT reagent Kit (Promega M-MLV M1705, Madison, USA). qRT‐PCR using SYBR Green Mix (TAKARA, Dalian, China) was carried out on Roche LightCycler 480II system in triplicate. Primers for GABPB1-AS1 and β-actin were synthesized by Genechem Co., Ltd. (Shanghai, China). The mRNA expressions were normalized to β-actin. The expressing levels of lncRNAs were defined based on the threshold cycle (Ct), and calculated using the 2-∆∆CT method. The primers were as follows:

- GABPB1-AS1 forward primer:CAACTAGGCAGACTGGGACG,
- GABPB1-AS1 reverse primer:AGGTGGCAGTAATCCAAGCA,
- β-actin forward primer:GCGTGACATTAAGGAGAAGC,
- β-actin reverse primer:CCACGTCACACTTCATGATGG.

### Statistical analysis

For the differential expression analysis, the screening criteria of the three kinds of dysregulated RNAs were as follows: adj. *p* value < 0.05 and |log2fold change|> 1.

For functional enrichment analysis, a *p* value of less than 0.05 was identified as having statistical significance.

Survival curves were plotted using the Kaplan–Meier method and the log‐rank test.

For qRT-PCR analysis, all data collected from three independent experiments were presented as mean ± standard deviation. Student's t test for comparison between two groups was performed for statistical analysis using SPSS 13.0 software (SPSS, Inc., Chicago, IL, USA). A p value of less than 0.05 was identified as having statistical significance.

## Data Availability

The datasets generated and/or analyzed during the current study are available in the NCBI GEO repository, https://www.ncbi.nlm.nih.gov/geo/query/acc.cgi?acc=GSE142699, ^68^. https://www.ncbi.nlm.nih.gov/geo/query/acc.cgi?acc=GSE142698, ^69^^.^ https://www.ncbi.nlm.nih.gov/geo/query/acc.cgi?acc=GSE103828^70^.

## Abbreviations

CN-AML: Cytogenetically normal acute myeloid leukemia
lncRNA: Long non-coding RNA
ceRNA: Competing endogenous RNA
MREs: MiRNA response elements
NCBI GEO: National Center for Biotechnology Information Gene Expression Omnibus
DEmRNAs: Differentially expressed mRNAs
DEmiRNAs: Differentially expressed miRNAs
DElncRNAs: Differentially expressed lncRNAs
GO: Gene Ontology
KEGG: Kyoto Encyclopedia of Genes and Genomes
RT: Reverse transcription
qRT-PCR: Quantitative real‐time polymerase chain reaction, CT: cycle threshold
OS: Overall survival

## References

[1] Antar AI, Otrock ZK, Jabbour E, Mohty M, Bazarbachi A (2020). FLT3 inhibitors in acute myeloid leukemia: Ten frequently asked questions. Leukemia.

[2] Mannelli F, Ponziani V, Bencini S, Bonetti MI, Benelli M, Cutini I, Gianfaldoni G, Scappini B, Pancani F, Piccini M (2017). CEBPA-double-mutated acute myeloid leukemia displays a unique phenotypic profile: A reliable screening method and insight into biological features. Haematologica.

[3] Chen X, Yan CC, Luo C, Ji W, Zhang Y, Dai Q (2015). Constructing lncRNA functional similarity network based on lncRNA-disease associations and disease semantic similarity. Sci. Rep..

[4] Lee JT (2012). Epigenetic regulation by long noncoding RNAs. Science.

[5] Nagano T, Fraser P (2011). No-nonsense functions for long noncoding RNAs. Cell.

[6] Wilusz JE, Sunwoo H, Spector DL (2009). Long noncoding RNAs: Functional surprises from the RNA world. Genes Dev..

[7] Chen X, Yan CC, Zhang X, You ZH (2017). Long non-coding RNAs and complex diseases: From experimental results to computational models. Brief. Bioinform..

[8] Chen X, Sun YZ, Guan NN, Qu J, Huang ZA, Zhu ZX, Li JQ (2019). Computational models for lncRNA function prediction and functional similarity calculation. Brief. Funct. Genom..

[9] Zhao C, Wang S, Zhao Y, Du F, Wang W, Lv P, Qi L (2019). Long noncoding RNA NEAT1 modulates cell proliferation and apoptosis by regulating miR-23a-3p/SMC1A in acute myeloid leukemia. J. Cell. Physiol..

[10] Garzon R, Volinia S, Papaioannou D, Nicolet D, Kohlschmidt J, Yan PS, Mrozek K, Bucci D, Carroll AJ, Baer MR (2014). Expression and prognostic impact of lncRNAs in acute myeloid leukemia. Proc. Natl. Acad. Sci. USA.

[11] Salmena L, Poliseno L, Tay Y, Kats L, Pandolfi PP (2011). A ceRNA hypothesis: The Rosetta Stone of a hidden RNA language?. Cell.

[12] Wang J, Liu ZH, Yu LJ (2019). Long non-coding RNA LINC00641 promotes cell growth and migration through modulating miR-378a/ZBTB20 axis in acute myeloid leukemia. Eur. Rev. Med. Pharmacol. Sci..

[13] Tian YJ, Wang YH, Xiao AJ, Li PL, Guo J, Wang TJ, Zhao DJ (2019). Long noncoding RNA SBF2-AS1 act as a ceRNA to modulate cell proliferation via binding with miR-188-5p in acute myeloid leukemia. Artif. Cells Nanomed. Biotechnol..

[14] Cheng Y, Su Y, Wang S, Liu Y, Jin L, Wan Q, Liu Y, Li C, Sang X, Yang L (2020). Identification of circRNA–lncRNA–miRNA–mRNA competitive endogenous RNA network as novel prognostic markers for acute myeloid leukemia. Genes (Basel).

[15] Zhang N, Chen Y, Shen Y, Lou S, Deng J (2019). Comprehensive analysis the potential biomarkers for the high-risk of childhood acute myeloid leukemia based on a competing endogenous RNA network. Blood Cells Mol. Dis..

[16] Yin X, Huang S, Zhu R, Fan F, Sun C, Hu Y (2018). Identification of long non-coding RNA competing interactions and biological pathways associated with prognosis in pediatric and adolescent cytogenetically normal acute myeloid leukemia. Cancer Cell Int..

[17] Han JD, Bertin N, Hao T, Goldberg DS, Berriz GF, Zhang LV, Dupuy D, Walhout AJ, Cusick ME, Roth FP, Vidal M (2004). Evidence for dynamically organized modularity in the yeast protein-protein interaction network. Nature.

[18] Herschbein L, Liesveld JL (2018). Dueling for dual inhibition: Means to enhance effectiveness of PI3K/Akt/mTOR inhibitors in AML. Blood Rev..

[19] Liu X, Ye Q, Zhao XP, Zhang PB, Li S, Li RQ, Zhao XL (2019). RAS mutations in acute myeloid leukaemia patients: A review and meta-analysis. Clin. Chim. Acta.

[20] Feng Y, Li L, Du Y, Peng X, Chen F (2020). E2F4 functions as a tumour suppressor in acute myeloid leukaemia via inhibition of the MAPK signalling pathway by binding to EZH2. J. Cell. Mol. Med..

[21] Jiang M, Chen Y, Deng L, Luo X, Wang L, Liu L (2019). Upregulation of SPAG6 in myelodysplastic syndrome: Knockdown inhibits cell proliferation via AKT/FOXO signaling pathway. DNA Cell Biol..

[22] Yang H, Zhang X, Cai XY, Wen DY, Ye ZH, Liang L, Zhang L, Wang HL, Chen G, Feng ZB (2017). From big data to diagnosis and prognosis: Gene expression signatures in liver hepatocellular carcinoma. PeerJ.

[23] Chang YH, Yu CH, Jou ST, Lin CY, Lin KH, Lu MY, Wu KH, Chang HH, Lin DT, Lin SW (2021). Targeted sequencing to identify genetic alterations and prognostic markers in pediatric T-cell acute lymphoblastic leukemia. Sci. Rep..

[24] Li B, Hu J, He D, Chen Q, Liu S, Zhu X, Yu M (2020). PPM1D knockdown suppresses cell proliferation, promotes cell apoptosis, and activates p38 MAPK/p53 Signaling pathway in acute myeloid leukemia. Technol. Cancer Res. Treat..

[25] Grimwade D, Hills RK, Moorman AV, Walker H, Chatters S, Goldstone AH, Wheatley K, Harrison CJ, Burnett AK, National Cancer Research Institute Adult Leukaemia Working G (2010). Refinement of cytogenetic classification in acute myeloid leukemia: Determination of prognostic significance of rare recurring chromosomal abnormalities among 5876 younger adult patients treated in the United Kingdom Medical Research Council trials. Blood.

[26] Zheng Q, Gu X, Yang Q, Chu Q, Dai Y, Chen Z (2021). DLX6-AS1 is a potential biomarker and therapeutic target in cancer initiation and progression. Clin. Chim. Acta.

[27] Song X, Cao G, Jing L, Lin S, Wang X, Zhang J, Wang M, Liu W, Lv C (2014). Analysing the relationship between lncRNA and protein-coding gene and the role of lncRNA as ceRNA in pulmonary fibrosis. J. Cell. Mol. Med..

[28] Liquori A, Ibanez M, Sargas C, Sanz MA, Barragan E, Cervera J (2020). Acute promyelocytic leukemia: A constellation of molecular events around a single PML-RARA fusion gene. Cancers (Basel).

[29] Ding ZX, Shen HJ, Miao JC, Chen SN, Qiu QC, Qi XF, Jin ZM, Wu DP, He J (2012). C-kit, NPM1 and FLT3 gene mutation patterns and their prognostic significance in 656 Chinese patients with acute myeloid leukemia. Zhonghua Xue Ye Xue Za Zhi.

[30] Ni ZF, Ma LJ, Shi LL, Shen PL, Zhao JQ (2021). Clinical characteristics of acute myeloid leukemia patients with RUNX1 gene mutation. Zhongguo Shi Yan Xue Ye Xue Za Zhi.

[31] Penny GD, Kay GF, Sheardown SA, Rastan S, Brockdorff N (1996). Requirement for Xist in X chromosome inactivation. Nature.

[32] Yang Z, Jiang X, Jiang X, Zhao H (2018). X-inactive-specific transcript: A long noncoding RNA with complex roles in human cancers. Gene.

[33] Schouten PC, Vollebergh MA, Opdam M, Jonkers M, Loden M, Wesseling J, Hauptmann M, Linn SC (2016). High XIST and low 53BP1 expression predict poor outcome after high-dose alkylating chemotherapy in patients with a BRCA1-like breast cancer. Mol. Cancer Ther..

[34] Sun W, Zu Y, Fu X, Deng Y (2017). Knockdown of lncRNA-XIST enhances the chemosensitivity of NSCLC cells via suppression of autophagy. Oncol. Rep..

[35] Wang C, Li L, Li M, Wang W, Liu Y, Wang S (2020). Silencing long non-coding RNA XIST suppresses drug resistance in acute myeloid leukemia through down-regulation of MYC by elevating microRNA-29a expression. Mol. Med..

[36] Cheng Z, Luo C, Guo Z (2020). LncRNA-XIST/microRNA-126 sponge mediates cell proliferation and glucose metabolism through the IRS1/PI3K/Akt pathway in glioma. J. Cell. Biochem..

[37] Ma L, Zhou Y, Luo X, Gao H, Deng X, Jiang Y (2017). Long non-coding RNA XIST promotes cell growth and invasion through regulating miR-497/MACC1 axis in gastric cancer. Oncotarget.

[38] Qin J, Bao H, Li H (2018). Correlation of long non-coding RNA taurine-upregulated gene 1 with disease conditions and prognosis, as well as its effect on cell activities in acute myeloid leukemia. Cancer Biomark..

[39] Wang X, Zhang L, Zhao F, Xu R, Jiang J, Zhang C, Liu H, Huang H (2018). Long non-coding RNA taurine-upregulated gene 1 correlates with poor prognosis, induces cell proliferation, and represses cell apoptosis via targeting aurora kinase A in adult acute myeloid leukemia. Ann. Hematol..

[40] Li G, Zheng P, Wang H, Ai Y, Mao X (2019). Long non-coding RNA TUG1 modulates proliferation, migration, and invasion of acute myeloid leukemia cells via regulating miR-370-3p/MAPK1/ERK. OncoTargets Ther..

[41] Li Q, Song W, Wang J (2019). TUG1 confers Adriamycin resistance in acute myeloid leukemia by epigenetically suppressing miR-34a expression via EZH2. Biomed. Pharmacother..

[42] Zang XJ, Li L, Du X, Yang B, Mei CL (2019). LncRNA TUG1 inhibits the proliferation and fibrosis of mesangial cells in diabetic nephropathy via inhibiting the PI3K/AKT pathway. Eur. Rev. Med. Pharmacol. Sci..

[43] Hui B, Xu Y, Zhao B, Ji H, Ma Z, Xu S, He Z, Wang K, Lu J (2019). Overexpressed long noncoding RNA TUG1 affects the cell cycle, proliferation, and apoptosis of pancreatic cancer partly through suppressing RND3 and MT2A. OncoTargets Ther..

[44] Li Q, Wang J (2020). LncRNA TUG1 regulates cell viability and death by regulating miR-193a-5p/Rab10 axis in acute myeloid leukemia. OncoTargets Ther..

[45] Wu X, Zheng X, Cheng J, Zhang K, Ma C (2020). LncRNA TUG1 regulates proliferation and apoptosis by regulating miR-148b/IGF2 axis in ox-LDL-stimulated VSMC and HUVEC. Life Sci..

[46] Tani H, Torimura M (2013). Identification of short-lived long non-coding RNAs as surrogate indicators for chemical stress response. Biochem. Biophys. Res. Commun..

[47] Tani H, Onuma Y, Ito Y, Torimura M (2014). Long non-coding RNAs as surrogate indicators for chemical stress responses in human-induced pluripotent stem cells. PLoS ONE.

[48] Qi W, Li Z, Xia L, Dai J, Zhang Q, Wu C, Xu S (2019). LncRNA GABPB1-AS1 and GABPB1 regulate oxidative stress during erastin-induced ferroptosis in HepG2 hepatocellular carcinoma cells. Sci. Rep..

[49] Gao S, Zhang F, Sun H, Yang X (2020). LncRNA GA-binding protein transcription factor subunit beta-1 antisense RNA 1 inhibits renal carcinoma growth through an MiR-1246/phosphoenolpyruvate carboxykinase 1 pathway. OncoTargets Ther..

[50] Ou R, Lv M, Liu X, Lv J, Zhao J, Zhao Y, Li X, Li W, Zhao L, Li J (2020). HPV16 E6 oncoprotein-induced upregulation of lncRNA GABPB1-AS1 facilitates cervical cancer progression by regulating miR-519e-5p/Notch2 axis. FASEB J..

[51] Suvanto M, Beesley J, Blomqvist C, Chenevix-Trench G, Khan S, Nevanlinna H (2020). SNPs in lncRNA regions and breast cancer risk. Front. Genet..

[52] Alkhateeb A, Rezaeian I, Singireddy S, Cavallo-Medved D, Porter LA, Rueda L (2019). Transcriptomics signature from next-generation sequencing data reveals new transcriptomic biomarkers related to prostate cancer. Cancer Inform..

[53] Zhang L, Yang P, Feng H, Zhao Q, Liu H (2021). Using network distance analysis to predict lncRNA–miRNA interactions. Interdiscip. Sci..

[54] Liu W, Jiang Y, Peng L, Sun X, Gan W, Zhao Q, Tang H (2021). Inferring gene regulatory networks using the improved Markov blanket discovery algorithm. Interdiscip. Sci..

[55] Chen X, Yan GY (2013). Novel human lncRNA-disease association inference based on lncRNA expression profiles. Bioinformatics.

[56] Chen X (2015). KATZLDA: KATZ measure for the lncRNA-disease association prediction. Sci. Rep..

[57] Zhang L, Liu T, Chen H, Zhao Q, Liu H (2021). Predicting lncRNA-miRNA interactions based on interactome network and graphlet interaction. Genomics.

[58] Robinson MD, McCarthy DJ, Smyth GK (2010). edgeR: A Bioconductor package for differential expression analysis of digital gene expression data. Bioinformatics.

[59] Li JH, Liu S, Zhou H, Qu LH, Yang JH (2014). starBase v2.0: Decoding miRNA–ceRNA, miRNA–ncRNA and protein-RNA interaction networks from large-scale CLIP-Seq data. Nucleic Acids Res..

[60] Wong N, Wang X (2015). miRDB: An online resource for microRNA target prediction and functional annotations. Nucleic Acids Res..

[61] Chou CH, Shrestha S, Yang CD, Chang NW, Lin YL, Liao KW, Huang WC, Sun TH, Tu SJ, Lee WH (2018). miRTarBase update 2018: A resource for experimentally validated microRNA-target interactions. Nucleic Acids Res..

[62] Fromm B, Billipp T, Peck LE, Johansen M, Tarver JE, King BL, Newcomb JM, Sempere LF, Flatmark K, Hovig E, Peterson KJ (2015). A uniform system for the annotation of vertebrate microRNA genes and the evolution of the human microRNAome. Annu. Rev. Genet..

[63] Jiang H, Ma R, Zou S, Wang Y, Li Z, Li W (2017). Reconstruction and analysis of the lncRNA–miRNA–mRNA network based on competitive endogenous RNA reveal functional lncRNAs in rheumatoid arthritis. Mol. Biosyst..

[64] Shannon P, Markiel A, Ozier O, Baliga NS, Wang JT, Ramage D, Amin N, Schwikowski B, Ideker T (2003). Cytoscape: A software environment for integrated models of biomolecular interaction networks. Genome Res..

[65] da Huang W, Sherman BT, Lempicki RA (2009). Systematic and integrative analysis of large gene lists using DAVID bioinformatics resources. Nat. Protoc..

[66] Xie C, Mao X, Huang J, Ding Y, Wu J, Dong S, Kong L, Gao G, Li CY, Wei L (2011). KOBAS 2.0: A web server for annotation and identification of enriched pathways and diseases. Nucleic Acids Res..

[67] Tang Z, Kang B, Li C, Chen T, Zhang Z (2019). GEPIA2: An enhanced web server for large-scale expression profiling and interactive analysis. Nucleic Acids Res..

[68] Esa, E., Hashim, A. K., Zakaria, Z., Hassan, A. N., Yusoff, Y. M., Kamaluddin, N. R., Rahman, A. Z., Mohamed, E. H., Meng, C. K., Mohamed, R. *et al.* Co-expression of microRNA and mRNA in cytogenetically normal acute myeloid leukemia patients [miRNA]. https://www.ncbi.nlm.nih.gov/geo/query/acc.cgi?acc=GSE142699 (2019).

[69] Esa, E., Hashim, A. K., Zakaria, Z., Hassan, A. N., Yusoff, Y. M., Kamaluddin, N. R., Rahman, A. Z., Mohamed, E. H., Meng, C. K., Mohamed, R. *et al.* Co-expression of microRNA and mRNA in cytogenetically normal acute myeloid leukemia patients [mRNA]. https://www.ncbi.nlm.nih.gov/geo/query/acc.cgi?acc=GSE142698 (2019).

[70] Feng Y, Shen Y, Chen H, Wang X, Zhang R, Peng Y, Lei X, Liu T, Liu J, Gu L (2018). Expression profile analysis of long non-coding RNA in acute myeloid leukemia by microarray and bioinformatics. Cancer Sci..

